# Dynamic Contrast-Enhanced Magnetic Resonance Imaging for Measuring Perfusion in Pancreatic Ductal Adenocarcinoma and Different Tumor Grade: A Preliminary Single Center Study

**DOI:** 10.3390/diagnostics13030521

**Published:** 2023-01-31

**Authors:** Inga Zaborienė, Vestina Strakšytė, Povilas Ignatavičius, Giedrius Barauskas, Rūta Dambrauskienė, Kristina Žvinienė

**Affiliations:** 1 Department of Radiology, Lithuanian University of Health Sciences, LT-50161 Kaunas, Lithuania; 2 Department of Surgery, Lithuanian University of Health Sciences, LT-50161 Kaunas, Lithuania; 3 Department of Oncology and Hematology, Lithuanian University of Health Sciences, LT-50161 Kaunas, Lithuania

**Keywords:** pancreatic adenocarcinoma, tumor grade, dynamic contrast-enhanced MRI

## Abstract

Background: Dynamic contrast-enhanced magnetic resonance imaging is a noninvasive imaging modality that can supply information regarding the tumor anatomy and physiology. The aim of the study was to analyze DCE-MRI perfusion parameters in normal pancreatic parenchymal tissue and PDAC and to evaluate the efficacy of this diagnostic modality in determining the tumor grade. Methods: A single-center retrospective study was performed. A total of 28 patients with histologically proven PDAC underwent DCE-MRI; the control group enrolled 14 patients with normal pancreatic parenchymal tissue; the radiological findings were compared with histopathological data. The study patients were further grouped according to the differentiation grade (G value): well- and moderately differentiated and poorly differentiated PDAC. Results: The median values of K^trans^, k_ep_ and iAUC were calculated lower in PDAC compared with the normal pancreatic parenchymal tissue (*p* < 0.05). The mean value of Ve was higher in PDAC, compared with the normal pancreatic tissue (*p* < 0.05). K^trans^, k_ep_ and iAUC were lower in poorly differentiated PDAC, whereas Ve showed no differences between groups. Conclusions: Ve and iAUC DCE-MRI perfusion parameters are important as independent diagnostic criteria predicting the probability of PDAC; the Ktrans and iAUC DCE-MRI perfusion parameters may serve as effective independent prognosticators preoperatively identifying poorly differentiated PDAC.

## 1. Introduction

Pathological grading of pancreatic ductal adenocarcinoma (PDAC) might be obtained preoperatively by fine needle biopsy (FNB). This way, some patients with a disease unsuitable for upfront surgery might be identified, even if considered resectable by high-quality imaging. However, FNB is associated with various complications and might postpone the treatment. Endoscopic ultrasonography-guided tissue acquisition (EUS-TA) represents the criterion standard for the diagnostic evaluation of pancreatic masses. However, the relatively high rate of false negatives still represents a common pitfall associated with this procedure [1]. If a poorly differentiated or anaplastic tumor is histologically confirmed, therapy might be started with a non-surgical approach. After neoadjuvant therapy, patients could undergo surgical exploration if the disease remains stable or in cases of downstaging. Therefore, noninvasive identification of tumor grade would be preferred in terms of improved patient selection. This is an interesting topic, as grading of the tumor is a significant prognostic factor in patients suffering from PDAC. Identifying new biomarkers is preferred to move toward an individual treatment of the patient, while unnecessary treatment could be prevented in others. Radiologists’ knowledge of PDAC is based on morphological changes when performing imaging. Recently, minimal progress has been made in understanding the pathophysiology of PDAC via different imaging modalities, one of which is perfusion computed tomography (CT) [2,3,4].

Without the hazard of ionizing radiation, dynamic contrast-enhanced magnetic resonance imaging (DCE-MRI) is a noninvasive imaging modality that can provide anatomical and physiological information of the tumor [5,6,7,8,9,10]. Preliminary studies supported the importance of magnetic resonance (MR) perfusion in the evaluation of abdominal organs [5,11,12,13,14]. The aim of the study was to analyze DCE-MRI perfusion parameters in normal pancreatic parenchymal tissue and PDAC and to estimate the efficacy of this diagnostic modality in determining the tumor grade. 

## 2. Materials and Methods

**Figure 1:**
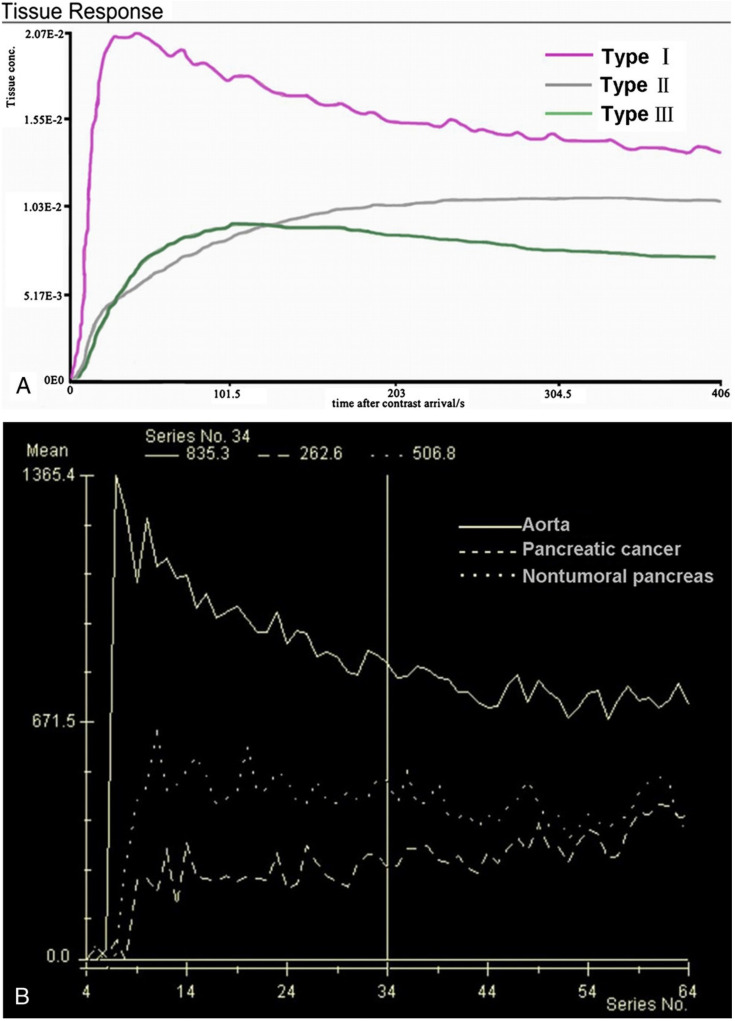
(**A**) SI-T curves of all nontumoral pancreatic tissue showed type I pattern enhancement. Pancreatic tumors showed type II or type III pattern enhancement; (**B**) Curve graph shows that pancreatic tumor enhances lesser than surrounding nontumoral pancreatic tissue. Time–signal–intensity curves selected for further data evaluation: type I, characterized by quick enhancement and quick washout; type II, with slow enhancement, which is followed by slow constant enhancement; type III, slow enhancement followed by slow washout [6].

We performed a retrospective single-center study in the Department of Radiology at the Hospital of Lithuanian University of Health Sciences Kaunas Clinics. The Regional Biomedical Research Ethics Committee approved the study (protocol No. BE-2-22). All the patients gave informed consent. Forty-four (44) patients with histologically proven PDAC underwent DCE-MRI from September 2019 to January 2021. The inclusion criteria were as follows: histologically proven pancreatic head PDAC; tumor ≥ 2.0 cm in size; estimated glomerular filtration rate (eGFR) > 30 mL/min; absence of contraindications to MR examination. We included 28 subjects with pancreatic head PDAC. Perfusion qualitative and quantitative assessments were obtained in all cases. We excluded 16 patients who could not complete the examination because of pain or disagreed to participate in the study. The inclusion criteria for the control group patients were as follows: no history of abdominal disorders, no significant medical history of diabetes or other pancreatic disorders, no cystic or benign liver and cystic kidney lesions. In total, 14 patients with non-tumorous pancreatic tissue were included as controls. MRI was performed using a 1.5T MR system (Siemens Magnetom Avanto, Siemens Healthineers, Erlangen, Germany) in the supine position. Patients fasted for 6 to 8 h before the DCE-MRI scan. All DCE-MRI examinations were performed using an injection of 0.2 mL/kg Gd-based contrast media (Gadovist^®^, Bayer AG, Leverkusen, Germany), at a rate of 3.0 mL/s, followed by 20 mL of saline at the same injection rate. All the patients were instructed to breathe slowly during the examination. Two observers with 10 and 20 years of experience interpreting pancreatic MR images reviewed the original MR imaging data. A DCE-MRI postprocessing software program (Tissue 4D; Siemens Magnetom Avanto, Siemens Healthineers, Erlangen, Germany) was used to obtain both a time–signal–intensity curve (TSIC) and to generate perfusion maps for each healthy pancreatic parenchyma, tumor, and different tumor grade. Perfusion maps of the volume transfer coefficient (Ktrans), extracellular extravascular volume fraction (Ve), rate constant (K_ep_), and initial area under the concentration curve in 60 s (iAUC) were generated. T1 mapping was computed from the T1-weighted acquisitions with different flip angles. The region of interest (ROI) was drawn on the abdominal aorta to obtain an arterial input function. The other three ROIs in the head of the pancreas were selected. The area of each ROI was 20 to 40 mm^2^. The mean perfusion values of all the ROIs were calculated and used for further evaluation. The pancreatic duct, necrotic, or cystic areas of the tumor were avoided. The qualitative analysis was based on TSICs, which were classified based on their shape by two radiologists into different types. We selected types of curves from the study by Wanling Ma et al. as an example for further evaluation (Figure 1) [6].

**Figure 2:**
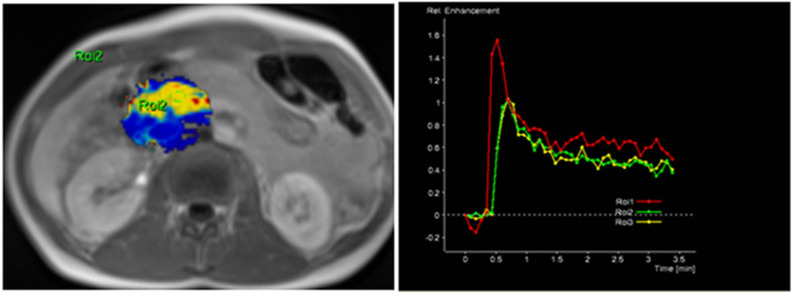
MRI perfusion images of non-tumorous pancreatic parenchymal tissue in the head of the pancreas with TSIC—Type I (characterized by fast enhancement and fast washout). Type I pattern enhancement was found in all tissues from control group patients.

**Figure 3:**
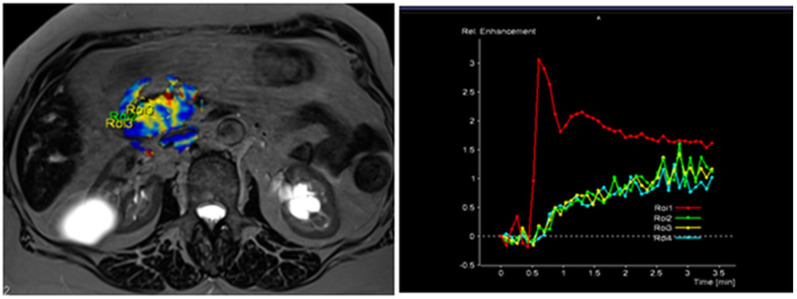
MRI perfusion images of G3 tumor in the head of the pancreas with TSIC—type II (slow enhancement and then continuous enhancement). The curve graph shows that pancreatic tumor enhances to a lesser extent than surrounding nontumoral pancreatic tissue. Most poorly differentiated (G3) tumors showed Type II pattern enhancement.

The radiological findings were compared with histopathological data. Histopathological analysis was performed at the Department of Pathological Anatomy, Lithuanian University of Health Sciences. The study patients were further grouped according to the differentiation grade (G value): well- and moderately differentiated (G1 + G2) and poorly differentiated (G3) PDAC. Images of DCE-MRI of non-tumorous pancreatic tissue and PDAC are presented in Figure 2 and Figure 3.

Statistical Analysis

We used the Kolmogorov–Smirnov and the Shapiro–Wilk tests to check data normality. Normally distributed data were expressed as mean values (SD—standard deviation), and abnormal distributed as medians. The Student’s *t*-test and Mann–Whitney U-test were used for normally and non-normally distributed data, respectively. We used discriminant function analysis to determine the differences between groups and between well-/moderately and poorly differentiated PDAC. 

We calculated cut-off points for best specificity, sensitivity, and accuracy, positive (PPV) and negative predictive values (NPV).

Statistical analysis was performed using ©SPSS for Windows 23.0 ™ software and ©Microsoft Excel 16™. Statistical significance was considered at *p*-value less than or equal to 0.05. 

## 3. Results

### 3.1. General Patient’s Characteristics

Radiological and histopathological data of 42 subjects were included. The control group enrolled 14 patients with normal pancreatic parenchymal tissue (Table 1). These patients underwent MRI due to other indications (IPMN—four subjects, benign liver lesions—seven subjects, cystic kidney lesions—three subjects). The mean age of patients was 60.64 (15.24) in this group. No difference in age or gender distribution between these groups was identified (*p* = 0.209 and *p* = 0.306, respectively, Table 1). 

### 3.2. Normal Pancreatic Parenchyma vs. Pancreatic Ductal Adenocarcinoma (PDAC)

The median K^trans^, k_ep_, and iAUC values and the mean value (SD) of V_e_ for the normal pancreatic parenchymal tissue were 0.178 (min^−1^), 0.861 (min^−1^), 16.457 (mmol/s), and 0.196 (0.114), respectively. The median values of K^trans^, k_ep_, and Iauc for PDAC patients were 0.106 (min^−1^), 0.406 (min^−1^), and 9.045 (mmol/s), respectively; the mean value (SD) of V_e_ for PDAC was 0.313 (0.169). 

The median values of K^trans^, k_ep_, and iAUC were calculated to be lower in PDAC compared with the normal pancreatic parenchymal tissue (*p* < 0.05). The mean value of Ve was higher in PDAC, compared with the normal pancreatic parenchymal tissue (*p* < 0.05). The data are presented in Table 2. 

We used the discriminant function analysis to determine differences between PDAC and the normal pancreatic parenchyma. All calculated MRI perfusion variables (Ktrans, Kep, Ve, and iAUC) were included to identify the most important ones. Cut-off points for all MRI perfusion parameters with their predictive values are presented in Table 3.

### 3.3. PDAC Independent Diagnostic Criteria

**Figure 4:**
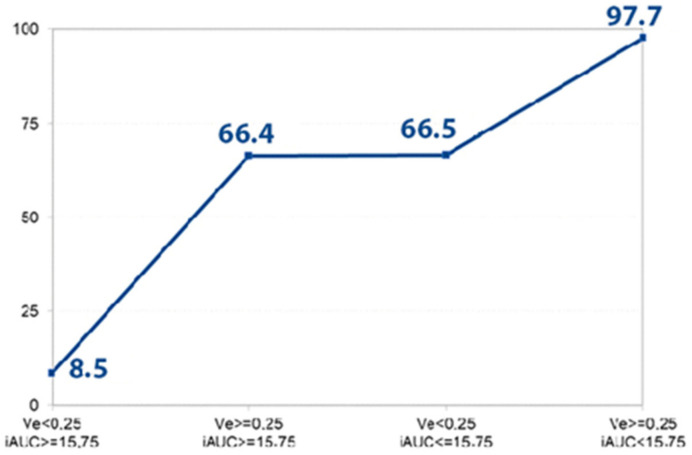
Disclosed probability for the PDAC. The graph shows the prognosticated probability of PDAC (%) (determined by logistic regression analysis) if the Ve and iAUC parameters or both of them exceed the defined cut-off value (Ve > 0.25, iAUC < 15.75). If both parameters are less than the determined cut-off point, the prognosticated probability for the presence of PDAC is 8.5%; if both values achieve the defined cut-off point, the estimated probability for the existence of PDAC is 97.7%.

The logistic regression model was used to disclose the independent diagnostic MRI perfusion criteria of PDAC. Four main parameters—Ktrans, Kep, Ve, and iAUC—were included in the stepwise analysis to determine the most significant ones (Table 4 and Figure 4), disclosing iAUC and Ve as the most important independent discriminators.

### 3.4. Well-/Moderately Differentiated (G1/G2) PDAC vs. Poorly Differentiated (G3) PDAC

There were 10 (35.7%) subjects with well-/moderately (G1 + G2), and 18 (64.3%) with poorly differentiated (G3) tumors. Analysis revealed that K^trans^, k_ep_, and iAUC were lower in poorly differentiated PDAC, whereas Ve showed no differences in G1 + G2 and G3 PDAC. The distribution between perfusion parameters is presented in Table 5.

We performed discriminant function analysis to identify differences between well-/moderately and poorly differentiated PDAC. Four main MRI perfusion variables (Ktrans, Kep, Ve, and iAUC) were included to establish the most important ones. 

The K^trans^ value less than 0.109 was concomitant to the presence of G3 PDAC with an 83.3% sensitivity and 90% specificity (AUC = 0.994); k_ep_ less than 0.344 was concomitant to the presence of PDAC with a 50% sensitivity and 90% specificity (AUC = 0.65); a higher than 0.272 Ve value was connected to the presence of G3 PDAC with a sensitivity of 44% and specificity of 30% (AUC = 0.428); an iAUC value less than 12.592 was concomitant to the presence of G3 PDAC with an 83.3% sensitivity and 90% specificity (AUC = 0.872). Cut-off points for all MRI perfusion parameters are presented in Table 6.

### 3.5. Independent Diagnostic Criteria of Poorly Differentiated (G3) PDAC

**Figure 5:**
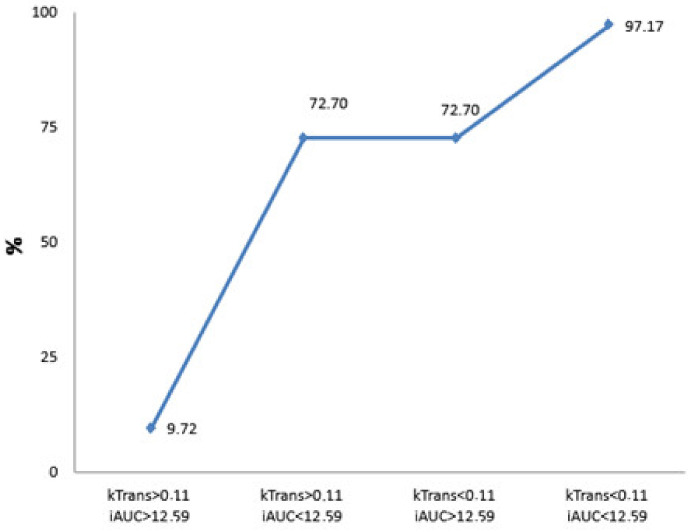
Calculated probability for the presence of poorly differentiated PDAC. The graph shows the prognosticated probability of G3 PDAC (%) if the Ktrans and iAUC parameters or both of them exceed the defined cut-off value (Ktrans < 0.11, iAUC < 12.59). If both parameters are less than the determined cut-off point, the prognosticated probability for the presence of G3 PDAC is 9.72%; if both values exceed the defined cut-off point, the estimated probability for the existence of PDAC is 97.17%.

The logistic regression model was chosen to disclose the independent diagnostic MRI perfusion criteria of poorly differentiated PDAC. Four main parameters—Ktrans, Kep, Ve, and iAUC—were included in the stepwise analysis to determine which of them were most important (Table 7 and Figure 5), identifying Ktrans and iAUC as significant independent discriminators.

## 4. Discussion

PDAC is a very heterogeneous tumor type; therefore, patients diagnosed with the same tumor stage often have markedly different clinical prognoses [15,16,17]. Diffusion-weighted imaging (DWI) with the apparent diffusion coefficient (ADC) predicts tumor grade and prognosis of various abdominal neoplasms, such as pancreatic neuroendocrine tumor, gastrointestinal tumors (GIST), rectal cancer, and hepatocellular carcinoma (HCC) [12,18,19,20,21].

The present study shows the significance of setting the role and value of the DCE-MRI analysis in diagnosing PDAC and evaluating the tumor grade without any ionizing radiation exposure for the patient. It is a noninvasive technique that can provide anatomical and physiological information about the tumor. DCE-MRI has been used to investigate microcirculation and microvasculature in different organs quantitatively. We analyzed the importance of MRI perfusion parameters in foreseeing PDAC and different tumor grades [4].

Another study of gallbladder cancer by Ji Hye Min et al. found the tumor ADC cut-off value to be an independent prognostic factor that ensures long-term disease-free survival (DFS) [16]. According to the literature, the differentiation grade of the tumor is a significant outcome prognosticator in PDAC patients [22]. Patients with low-grade (well-differentiated) PDAC have better survival rates than those with poorly differentiated PDAC [15,23,24,25,26]. Shibata K et al. reported that undifferentiated PDAC strongly predicts poor outcomes since it is related to hepatic metastases [15]. Tumor size and lymph node metastases influence survival less than tumor grade. Therefore, a noninvasive imaging modality able to predict a differentiation grade of PDAC before surgery would help to identify the aggressive prognosis of G3 tumors. This can allow optimizing therapeutic strategies and improve survival. Wasif N et al. in their study proposed the ability to include the tumor grade in PDAC staging—a novel TNMG staging system [10].

We agree with Facciorusso A et al., who recently confirmed contrast-enhanced harmonic endoscopic ultrasound (CH-EUS) as a helpful tool to identify the ideal target area for EUS-FNA avoiding the anechoic areas and vessels inside the tumor [27]. They also reported EUS-guided TA as a valuable tool in the diagnostic algorithm of pancreatic masses; however, this method is still impaired by the relatively high rate of false negative results, mainly due to inadequate tissue samples. Most of these false negative cases are related to the presence of fibrosis and necrosis inside the tumor, thus usually requiring several needle passes to achieve adequate samples. Contrast enhancement was found to be able to properly characterize the areas of necrosis as well as the vessels. Therefore, this tool might help to identify the target area for FNA, thus decreasing the rate of false negative results by avoiding the areas rich in blood, necrosis, and fibrosis. Still, this method remains invasive.

Noninvasive preoperative identification of poorly differentiated PDAC may potentially define patients who may benefit from preoperative biopsy followed by neoadjuvant therapy for morphologically confirmed poorly differentiated PDAC or who may be enrolled in further clinical trials. In this way, patients might be eligible for individualized treatment.

In our previous study, we reported the significance of perfusion CT and MRI DWI in estimating poorly differentiated PDAC [13]. Still, one of the most significant disadvantages of perfusion CT remains a high radiation dose.

The qualitative assessment based on the signal intensity per time showed similar results to other studies reported in the literature [7,28,29,30,31]. All patients with non-tumorous pancreatic tissue showed a TSIC-shape 1. All PDAC patients showed a TSIC-shape 2, characterized by slow progressive enhancement. TSIC did not differ between different tumor grades because of overlap in the imaging features. The perfusion analysis showed a difference between Ktrans, Ve, Kep, and iAUC values obtained in non-tumorous pancreatic tissue, PDAC, and different tumor grade.

The K^trans^, k_ep_, and iAUC values of PDAC (0.106 min^−1^, 0.406 min^−1^, and 9.045 mmol/s, respectively) were lower than those of non-tumorous pancreatic tissue (0.178 min^−1^, 0.861 min^−1^, and 16.457 mmol/s, respectively). Moreover, all these perfusion values were significantly lower in G3 PDAC (0.059 min^−1^, 0.357 min^−1^, and 5.202 mmol/s, respectively). The Ktrans and Kep values reflect blood flow; thus, the described results agreed with the post-contrast behavior of the PDAC. According to Jin Xu et al., PDAC with poor blood supply had lower blood volume and blood flow when compared with normal pancreatic parenchyma. Therefore, more connective tissue and fibrous tissue proliferation in PDAC leads to the higher vascular pressure of EES, which further reduces the value of K^trans^ [32]. 

Jae Hyun Kim et al. [8] in their study also reported these values to be significantly lower in PDAC (0.042 min^−1^, 0.761 min^−1^, and 2.841 mmol/s) when compared with non-tumorous pancreatic tissue (0.387 min^−1^, 6.376 min^−1^, and 7.156 mmol/s). The authors assumed that these findings are predictable in regions where permeability is high compared with blood flow (in PDAC), and that Ktrans represents tissue blood flow as well as iAUC [8]. Significantly low Ktrans and iAUC values of PDAC correspond with the fact that pancreatic cancer is a hypovascular tumor [8]. Further, our results coincide with the results of pancreatic perfusion CT. We strongly believe that MRI perfusion parameters reflect the vascular physiology of solid hypovascular pancreatic tumors similar to perfusion CT parameters [2,13,32].

We also found a significant difference of Ktrans, Kep, and iAUC values between well-/moderately (G1/2) and poorly differentiated (G3) PDAC (0.175 min^−1^, 0.521 min^−1^, and 15.6 mmol/s and 0.059 min^−1^, 0.357 min^−1^, and 5.202 mmol/s respectively). These results are similar to the CT perfusion parameters calculated in our previous studies [2,13]. Ktrans and iAUC perfusion parameters were also significant independent discriminators for G3 PDAC. One of the strongest parts of our study is that none of the previously mentioned studies evaluated any MRI perfusion parameters for estimating poorly differentiated tumors. Only Wanling Ma reported some data about perfusion parameters and correlation with fibrotic tissue [6]. He found a negative correlation between K^trans^ of PDAC and fibrosis content and positive correlation of fibrosis to Ve. Yao X et al. also found statistically different values of K^trans^ between normal pancreatic tissue and PDAC. Calculations of perfusion parameters between different tumor grades were also not included [32]. 

Our study showed that there were significant differences in MRI perfusion parameters between non-tumorous pancreatic tissue and PDAC, as well as different tumor grades.

The differentiation between G1/2 and G3 PDAC during imaging is impossible. This confusion may lead to surgical treatment of G3 disease. Therefore, remarkably different perfusion parameters may provide helpful information for decision making. Our data demonstrate the potential role of DCE-MRI in the preoperative detection of high-grade tumors. Furthermore, the defined cut-off values of MRI perfusion parameters were found to be independent prognosticators for the presence of poorly differentiated PDAC. 

If Ve and iAUC values exceeded the determined cut-off point, the estimated probability for the presence of PDAC reached almost 100% in this study. Therefore, estimated Ve and iAUC parameters may serve as promising independent diagnostic criteria predicting the probability of PDAC. Moreover, using the combination of Ktrans and iAUC values, the estimated probability for the presence of high-grade PDAC reached 97%.

Our study has some limitations. Firstly, a low number of patients were included in this retrospective study. Additionally, this was a single-center study performed with a specific MRI machine and software package for calculation of tissue perfusion parameters. To show the validity of our data, further standardized perfusion MRI protocols and multicenter studies are needed. 

Finally, a control group of patients with a histologically proven diagnosis of chronic pancreatitis to evaluate the difference in DCE-MRI perfusion parameters would be of great value in establishing the reliability of DCE-MRI diagnosing PDAC.

## 5. Conclusions

- The estimated Ve and iAUC DCE-MRI perfusion parameters are important as independent diagnostic criteria predicting the probability of PDAC.
- If Ve and iAUC values are combined, the estimated probability for the presence of PDAC reaches almost 100%.
- The Ktrans and iAUC DCE-MRI perfusion parameters may be effective independent prognosticators preoperatively estimating poorly differentiated PDAC.
- If Ktrans and iAUC values are combined, the estimated probability for the presence of high-grade PDAC reaches 97%.

## Figures and Tables

**Table 1:** The distribution between gender of PDAC and control group.

	Non-Tumorous Pancreatic Tissue (*n* = 14) Mean (SD) or N (%)	PDAC (*n* = 28) Mean (SD) or N (%)	*p* Value
Age	60.64 (15.24)	66.46 (15.24)	=0.209
Female	2 (14.3%)	8 (28.6%)	=0.306
Male	12 (85.7%)	20 (71.4%)	=0.306
Total	14 (100%)	28 (100%)	

Values are mean (standard deviation (SD)).

**Table 2:** DCE-MRI perfusion parameters in normal pancreatic parenchymal tissue and PDAC.

Parameters	Non-Tumorous Pancreatic Tissue Mean (SD) or Median * (q1–q3) Value	PDAC Mean (SD) or Median * (q1–q3) Value	*p* Value
K^trans^ * (min^−1^)	0.178 (0.0295–0.538)	0.106 (0.0298–0.538)	=0.033
k_ep_ * (min^−1^)	0.861 (0.519–3.035)	0.406 (0.199–1.054)	=0.006
V_e_	0.196 (0.114)	0.313 (0.169)	=0.012
iAUC * (mmol/s)	16.457 (11.23–29.613)	9.045 (3.309–15.452)	=0.005

* Median in abnormal distribution of parameters. Abbreviations: SD—standard deviation; K^trans^—volume transfer coefficient; Ve—extracellular extravascular volume fraction; K_ep_—rate constant; iAUC—initial area under the concentration curve in 60 s.

**Table 3:** Average of Ktrans, Kep, Ve, and iAUC in the presence of PDAC.

PDAC	K^trans^	k_ep_	V_e_	iAUC
AUC	0.704	0.765	0.689	0.768
Cut-off point	<0.19	≤0.4	≥0.25	<15.75
Sensitivity	92.9	50.0	53.6	64.3
Specificity.	50.0	92.9	92.9	82.8
PPV	78.8	93.3	93.8	82.8
NPV	77.8	48.1	50.0	64.3

Abbreviations: PPV—positive predictive value; NPV—negative predictive value; ROC—receiver operating characteristic; K^trans^—volume transfer coefficient; Ve—extracellular extravascular volume fraction_;_ K_ep_—rate constant; iAUC—initial area under the concentration curve in 60 s.

**Table 4:** Logistic regression for disclosing the probability occurrence of PDAC.

	B	S.E.	*p* Value	Exp (B)	95% C.I. for EXP (B) Lower	95% C.I. for EXP (B) Upper
iAUC (mmol/s)	3.068	1.145	0.007	21.5	2.28	202.778
V_e_	3.061	1.209	0.011	21.354	1.997	228.318

Abbreviations: Ve—extracellular extravascular volume fraction; iAUC—initial area under the concentration curve in 60 s.

**Table 5:** DCE-MRI perfusion parameters in different grades of PDAC.

Parameters	Mean (SD) or Median * (q1–q3) Value (G1 + G2), (*n* = 10)	Mean (SD) or Median * (q1–q3) Value (G3), (*n* = 18)	*p* Value
K^trans^ * (min^−1^)	0.175 (0.132–0,182)	0.059 (0.034–0.106)	=0.020
k_ep_ * (min^−1^)	0.521 (0.369–1.091)	0.357 (0.165–0.623)	<0.001
V_e_	0.335 (0.139)	0.300 (0.185)	=0.254
iAUC * (mmol/s)	15.600 (14.461–17.598)	5.202 (1.771–10.712)	=0.035

* Median in abnormal distribution of parameters; abbreviations: SD—standard deviation; K^trans^—volume transfer coefficient; Ve—extracellular extravascular volume fraction; K_ep_—rate constant; iAUC—initial area under the concentration curve in 60 s.

**Table 6:** Analysis of the poorly differentiated (G3) PDAC.

Poorly Differentiated (G3) PDAC	K^trans^	k_ep_	V_e_	iAUC
AUC	0.994	0.65	0.428	0.872
Cut-off point	≤0.109	≤0.344	≥0.272	≤12.592
Sensitivity	83.3	50	44	83.3
Specificity.	90	90	30	90
PPV	94	90	53	94
NPV	75	50	23	75

Abbreviations: PPV—positive predictive value; NPV—negative predictive value; ROC—receiver operating characteristic; K^trans^—volume transfer coefficient; Ve—extracellular extravascular volume fraction; K_ep_—rate constant; iAUC—initial area under the concentration curve in 60 s.

**Table 7:** Logistic regression for estimating the probability occurrence of poorly differentiated (G3) PDAC.

	B	S.E.	*p* Value	Exp (B)	95% C.I. for EXP (B) Lower	95% C.I. for EXP (B) Upper
Ktrans	3.807	1.229	0.002	45	4.044	500.693
iAUC	3.281	1.462	0.025	26.599	1.516	466.594

Abbreviations: Ktrans—volume transfer coefficient; iAUC—initial area under the concentration curve in 60 s.

## Data Availability

Not applicable.
